# Impact of glucometabolic status on type 4a myocardial infarction in patients with non–ST-segment elevation myocardial infarction: the role of stress hyperglycemia ratio

**DOI:** 10.1186/s12933-025-02837-y

**Published:** 2025-10-11

**Authors:** Matteo Armillotta, Luca Bergamaschi, Francesco Angeli, Marta Belmonte, Marcello Casuso Alvarez, Angelo Sansonetti, Damiano Fedele, Sara Amicone, Lisa Canton, Davide Bertolini, Andrea Impellizzeri, Francesca Bodega, Nicole Suma, Francesco Pio Tattilo, Daniele Cavallo, Ornella Di Iuorio, Khrystyna Ryabenko, Andrea Rinaldi, Francesco Saia, Gianni Casella, Jacopo Lenzi, Paola Rucci, Pasquale Paolisso, Carmine Pizzi

**Affiliations:** 1 Department of Medical and Surgical Sciences (DIMEC), Alma Mater Studiorum - University of Bologna, Bologna, Italy; 2 Cardiovascular Division, Morgagni-Pierantoni University Hospital, Via Carlo Forlanini 34, 47121 Forlì, Italy; 3 Department of Advanced Biomedical Sciences, University Federico II, Naples, Italy; 4 Cardiology Unit, Sant’Andrea University Hospital, Rome, Italy; 5 Cardiology Unit, IRCCS Azienda Ospedaliero-Universitaria di Bologna, Bologna, Italy; 6 Unit of Cardiology, Maggiore Hospital, Bologna, Italy; 7 Division of Hygiene and Biostatistics, Department of Biomedical and Neuromotor Sciences, Alma Mater Studiorum, University of Bologna, Bologna, Italy

**Keywords:** Admission blood glucose (ABG), Stress hyperglycemia ratio (SHR), Non-ST-elevation myocardial infarction (NSTEMI), Percutaneous coronary intervention (PCI), Periprocedural myocardial infarction, Type 4a myocardial infarction, Diabetes, Cardiovascular outcomes

## Abstract

**Background:**

Type 4a myocardial infarction (MI) is a relevant complication in non–ST-segment elevation myocardial infarction (NSTEMI) patients undergoing percutaneous coronary intervention (PCI). While glucometabolic status has been linked to type 4a MI in chronic coronary syndromes, data in the acute setting are lacking. This study aimed to assess the association of glucometabolic parameters—admission blood glucose (ABG), glycated hemoglobin (HbA1c) and stress hyperglycemia ratio (SHR)—with type 4a MI in NSTEMI patients undergoing PCI and evaluate their independent predictive role.

**Methods:**

Consecutive NSTEMI patients undergoing PCI from the AMIPE multicenter prospective registry (NCT03883711) with stable or falling pre-procedural cardiac troponin levels were analyzed. The optimal glucometabolic predictor of type 4a MI among ABG, HbA1c and SHR was identified using receiver operating characteristic analysis. The best cut-off for each parameter was derived using Youden’s index. Regression analysis and Kaplan–Meier curves were performed to identify independent predictors of type 4a MI and their prognostic implications.

**Results:**

The study population included 1005 patients (mean age 70.3 ± 12.5 years, 25.5% females), with 45.9% having diabetes mellitus. SHR showed a significantly higher accuracy (AUC 0.69, 95% CI 0.65–0.73) in predicting type 4a MI compared with ABG and HbA1c (*p* < 0.001), with an optimal cut-off of 1.14, consistent across diabetic and non-diabetic patients. SHR > 1.14 was independently associated with type 4a MI (aOR = 2.73; 95% CI 1.70–4.42; *p* < 0.001), unlike ABG and HbA1c, and was also linked to an increased risk of long-term major adverse cardiovascular events (*p* < 0.001).

**Conclusions:**

SHR emerged as a strong predictor of type 4a MI in NSTEMI patients undergoing PCI, outperforming other glucometabolic markers.

**Graphical Abstract:**

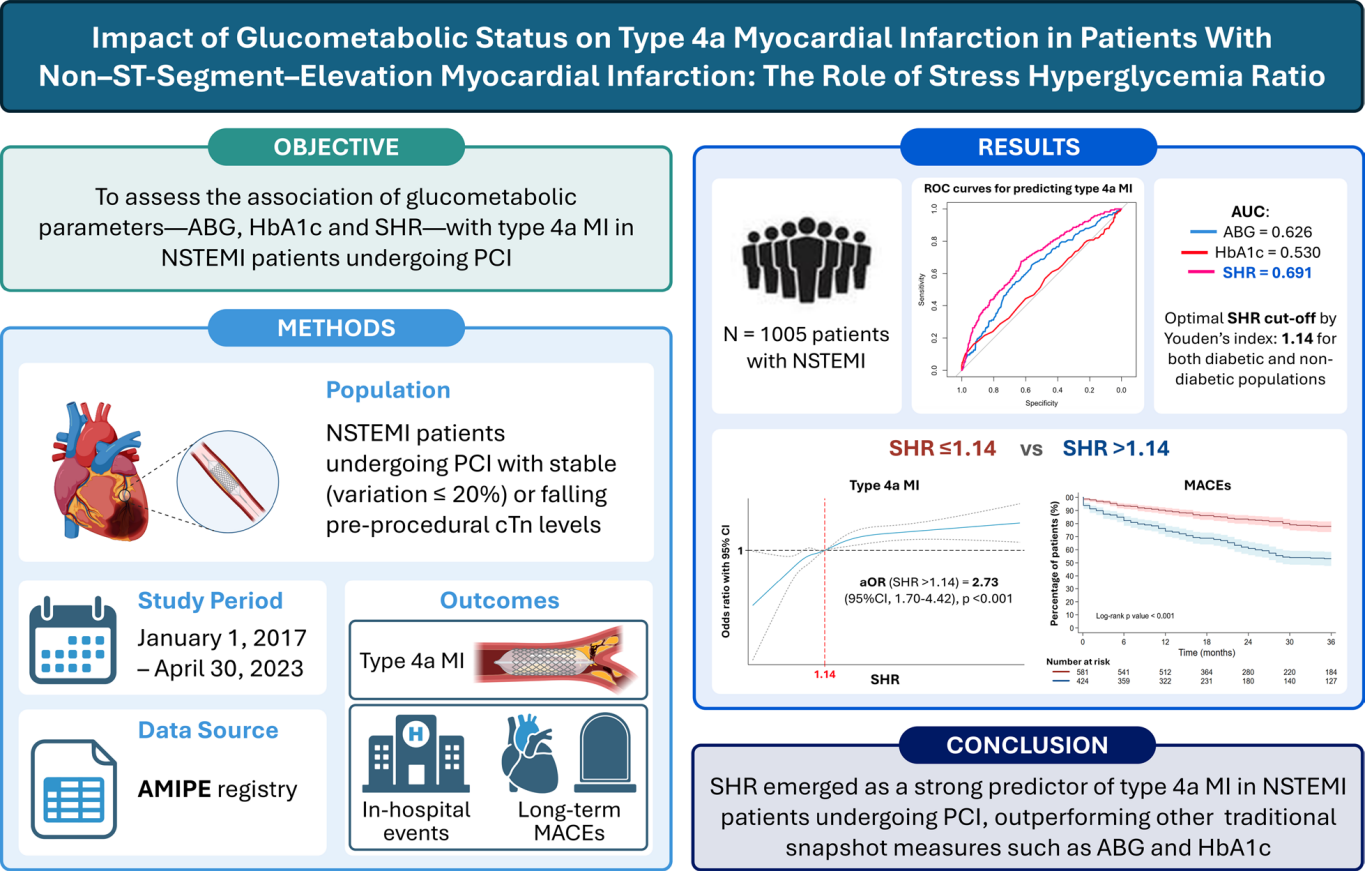

**Supplementary Information:**

The online version contains supplementary material available at 10.1186/s12933-025-02837-y.

## Research insights

**What is currently known about this topic?**

- Type 4a myocardial infarction (MI) is a serious complication in non–ST-segment elevation myocardial infarction (NSTEMI) patients undergoing percutaneous coronary intervention (PCI), associated with increased mortality and major adverse cardiovascular events. Although glucometabolic status has been linked to type 4a MI in chronic coronary syndromes, its role in the acute setting remains unknown.

**What is the key research question?**

- Can glucometabolic markers, specifically admission blood glucose (ABG), glycated hemoglobin (HbA1c), and stress hyperglycemia ratio (SHR), predict the occurrence of type 4a MI in patients with NSTEMI undergoing PCI?

**What is new?**

- This is the first study to assess the predictive value of glucometabolic status for type 4a MI in patients with NSTEMI. The SHR emerged as the most accurate glucometabolic marker for identifying patients at risk. An SHR greater than 1.14 was independently associated with a higher incidence of type 4a MI, regardless of diabetes status, and outperformed both ABG and HbA1c in risk prediction.

**How might this study influence clinical practice?**

- SHR could be easily integrated into routine risk assessment to identify NSTEMI patients at higher risk of type 4a MI and adverse outcomes. Further research is needed to assess whether targeted interventions in high-SHR patients can improve periprocedural outcomes and long-term prognosis.

## Introduction

Type 4a myocardial infarction (MI) is a clinically relevant complication occurring in approximately 17% of patients with non–ST-segment elevation myocardial infarction (NSTEMI) undergoing percutaneous coronary intervention (PCI). It is independently associated with increased 1-year risk of all-cause mortality and major adverse cardiovascular events (MACEs) [1].

Among patients with acute myocardial infarction (AMI), glucometabolic status has an impact on in-hospital and long-term outcomes, regardless of the diabetic status and coronary anatomy [2–6]. Several studies have shown that stress-induced hyperglycemia is associated with higher thrombus burden and increased likelihood of periprocedural complications in AMI patients undergoing PCI, such as stent thrombosis and slow flow or no-reflow [7–10]. Remarkably, stress hyperglycemia ratio (SHR) has emerged as a novel marker that accurately reflects the metabolic hyperglycemic state in AMI patients, irrespective of the presence of diabetes mellitus (DM) [11]. Elevated SHR was found to be an independent predictor of increased risk of all-cause mortality, MACEs, and higher thrombus burden during invasive coronary angiography (ICA) [10, 12–14].

Despite evidence linking glucometabolic status to the occurrence of type 4a MI in patients with chronic coronary syndromes undergoing PCI [15–17], its role in the acute setting remains poorly defined. Therefore, in this study, we aimed to elucidate the association between the glucometabolic status—admission blood glucose (ABG), glycated hemoglobin (HbA1c) and SHR—and the occurrence of type 4a MI in NSTEMI patients undergoing PCI, to identify the most accurate parameter for risk stratification and to assess its prognostic implications.

## Methods

### Study design and population

This study included consecutive NSTEMI patients undergoing PCI between January 1, 2017, and April 30, 2023, with stable (variation ≤ 20%) or falling pre-procedural cardiac troponin (cTn) levels [1, 18]. Data were obtained from the ongoing multicenter prospective observational registry, “AMIPE: Acute Myocardial Infarction, Prognostic and Therapeutic Evaluation” (NCT03883711), designed to assess the characteristics and outcomes of patients admitted with a diagnosis of AMI.

NSTEMI diagnosis followed the Fourth Universal Definition of Myocardial Infarction (4th UDMI), and patients were managed and treated in accordance with current guidelines [18, 19]. Serial measurements of cTn were conducted for all patients from hospital admission. Exclusion criteria for the current analysis were: (i) absence of serial cTn measurements; (ii) evidence of rising pre-PCI cTn levels (unstable, variation > 20%); (iii) missing data on ABG or HbA1c levels; (iv) lack of informed consent.

The study protocol was approved by the institutional review board (Registration Number: 600/2018/Oss/AOUBo) and adhered to the principles of the Declaration of Helsinki. All patients were informed about their participation in the study and provided consent for the anonymous publication of scientific data. Details regarding data collection can be found in the Supplementary Material under Extended Methods.

### Definition of glucometabolic status

Pre-existing DM was defined as a documented history of DM at the time of hospitalization, regardless of treatment approach. DM was newly diagnosed during hospitalization based on fasting plasma glucose levels ≥ 126 mg/dL or HbA1c ≥ 48 mmol/mol [20]. Patients without a history of DM and HbA1c < 48 mmol/mol were classified as non-diabetic [20].

ABG levels were measured using the first available venous blood samples as part of the standard evaluation. HbA1c was assessed during the initial routine blood test conducted upon admission to the Cardiology Department. The SHR was calculated as ABG divided by estimated average glucose level. The latter was calculated using the formula: estimated average glucose (mg/dL) = 28.7 × HbA1c (%) − 46.7 [21–23]. Accordingly, SHR was calculated as SHR = ABG/[28.7 × HbA1c(%) − 46.7] [11, 24]. Hyperglycemic patients with ABG > 200 mg/dL were treated with an insulin-glucose infusion to reduce blood glucose to 126–199 mg/dL within the first 24 h, followed by subcutaneous insulin injections [25, 26].

### Definition of type 4a MI

According to the 4th UDMI, type 4a MI was diagnosed in the presence of a post-PCI cTn elevation greater than five times the 99th percentile URL in patients with normal baseline values, or in the presence of a post-PCI cTn elevation greater than 20% with an absolute postprocedural value of at least five times the 99th percentile URL in patients with elevated baseline cTn who had stable (variation ≤ 20%) or falling cTn levels, plus one of the following elements: (1) new ischemic ECG changes; (2) development of new pathological Q waves; (3) imaging evidence of new loss of viable myocardium or new regional wall motion abnormality in a pattern consistent with an ischemic etiology; (4) angiographic findings consistent with a procedural flow-limiting complication such as coronary dissection, loss of a side branch, slow flow, thrombus, or distal embolization [18].

Type 4a MI was adjudicated by two independent experts who reviewed all clinical and instrumental information collected during hospitalization, blinded to glucometabolic variables. In cases of disagreement, a third referee provided the final decision [1].

### Follow-up and clinical outcomes

In-hospital events were evaluated, including in-hospital deaths (both cardiac and non-cardiac), the use of mechanical circulatory support (including intra-aortic balloon pump, Impella, and extracorporeal membrane oxygenation), length of hospital stay, and new-onset cardiac arrhythmias, defined as a composite of new-onset atrial fibrillation and new-onset sustained ventricular tachycardia and/or ventricular fibrillation.

After discharge, patients were followed up through outpatient visits and/or telephone contacts using a standardized questionnaire. MACEs included all-cause mortality, non-fatal reinfarction, unplanned revascularization, non-fatal ischemic stroke, and hospitalization for heart failure (HF). Additional details on outcome definitions are provided in the Supplementary Material under Extended Methods.

### Statistical analysis

Continuous variables were summarized using the mean ± SD or the median and the interquartile range (IQR), according to the normality of the frequency distribution, while categorical variables were summarized as absolute and percentage frequencies. The normality of the frequency distribution was assessed using Shapiro–Wilk’s test.

Receiver operating characteristic (ROC) curve analysis was conducted to identify which glucometabolic parameter, among ABG, HbA1c, and SHR, was the most accurate predictor of type 4a MI in the overall cohort and stratified by diabetes status. The areas under the curves were compared using DeLong’s test. The best cut-off, balancing sensitivity and specificity, was determined as the one maximizing Youden’s index (sensitivity + specificity − 1). The overall population was then divided into two groups based on the best cut-off of the most accurate glucometabolic predictor of type 4a MI. Between-group comparisons were carried out using Student’s t-test or the Mann–Whitney U test for continuous variables, and the chi-square test or Fisher’s exact test, as appropriate, for categorical variables. Univariate logistic regression analyses were performed to identify variables associated with type 4a MI. All variables with a *p* ≤ 0.1 in the univariate regressions were entered in the multivariable logistic regression analysis. Additionally, interaction effects between SHR and selected clinical variables (those with a *p* ≤ 0.1 in univariate analysis) were tested by including corresponding interaction terms in the multivariable model.

Restricted cubic spline was fitted to evaluate the non-linear relationship between glucometabolic parameters and type 4a MI, while Kaplan–Meier analysis with the log-rank test was conducted to compare the cumulative incidence of clinical events between groups stratified by the optimal cut-off identified through ROC analysis. Net reclassification improvement (NRI) and integrated discrimination improvement (IDI) were calculated to assess the incremental value in predicting type 4a MI. These analyses were performed only for glucometabolic variables that proved to have a significant predictive accuracy for type 4a MI in ROC analysis.

To strengthen the robustness of our findings, a sensitivity analysis was performed by excluding patients with ABG and HbA1c values below the 10th percentile and above the 90th percentile.

All analyses were performed using Stata version 17 (StataCorp LLC, College Station, TX, USA) and R statistical software version 4.2.1 (R Foundation for Statistical Computing, Vienna, Austria). The significance level was set to *p* < 0.05.

## Results

### Study population and glucometabolic status

Out of 1925 screened patients with NSTEMI undergoing PCI, 164 had unstable cTn levels pre-PCI, 29 unavailable serial cTn measurements, and 727 unavailable HbA1c (Supplementary Fig. 1). The final study population consisted of 1005 patients with NSTEMI treated with PCI who had stable or falling pre-procedural cTn levels, including 461 patients with DM (45.9%) and 544 without DM (54.1%). Supplementary Table 1 presents the baseline characteristics of the final study population compared with those of patients excluded due to missing HbA1c measurements.

**Fig. 1:**
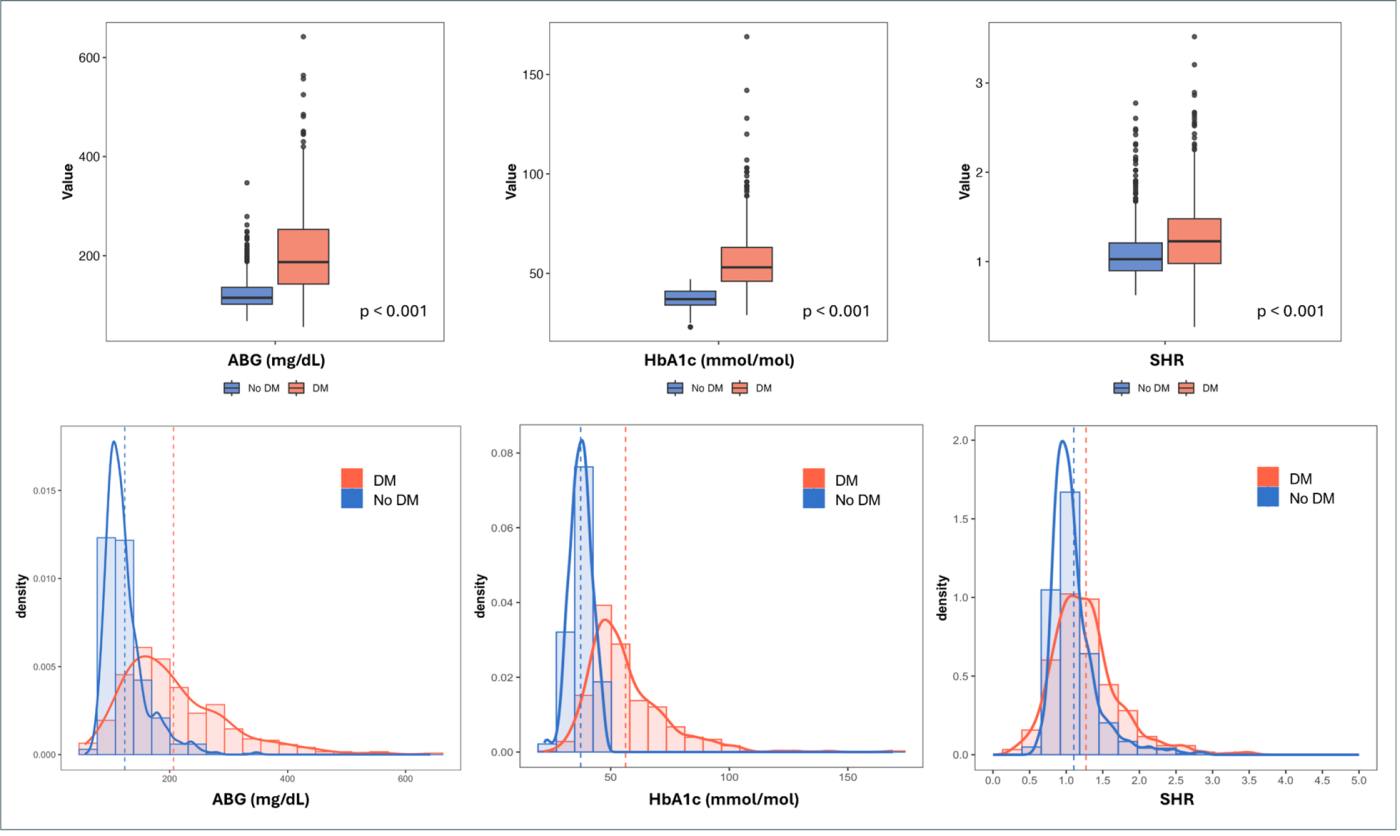
Admission glucometabolic profile of the overall study population, stratified by diabetes status. *DM* Diabetes mellitus; *ABG* Admission blood glucose; *HbA1c* Glycated hemoglobin; *SHR* Stress hyperglycemia ratio

The admission glucometabolic status of the study population, stratified by the presence or absence of DM, is shown in Fig. 1. Diabetic patients showed significantly higher ABG levels, HbA1c, and SHR (*p* < 0.001 for all).

**Fig. 2:**
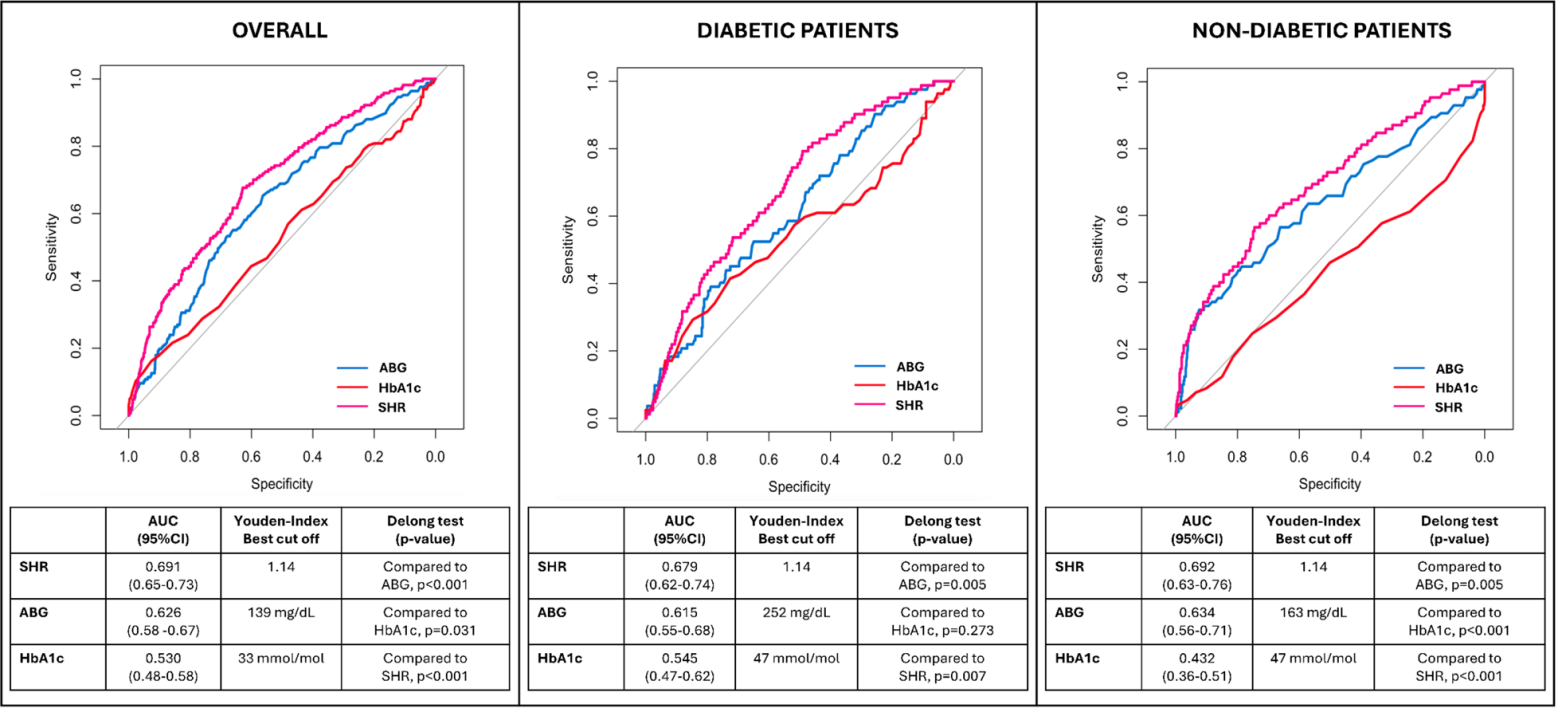
Comparison of ROC curves for predicting type 4a myocardial infarction using different glucometabolic parameters in the overall study population and separately for patients stratified by diabetes status. *ROC* Receiver operating characteristic; *AUC* Area under the curve; *ABG* Admission blood glucose; *HbA1c* Glycated hemoglobin; *SHR* Stress hyperglycemia ratio

ROC analysis showed that ABG and SHR were significantly associated with the occurrence of type 4a MI (*p* < 0.001 for both), whereas HbA1c was not (*p* = 0.252). In the overall study population, SHR demonstrated superior predictive performance for type 4a MI (AUC 0.691, 95% CI 0.65–0.73, *p* < 0.001, sensitivity 68%, specificity 63%) compared with ABG (AUC 0.626, 95% CI 0.58–0.67, *p* < 0.001, sensitivity 65%, specificity 56%, DeLong test *p* < 0.001). This finding was confirmed in both the diabetic and non-diabetic cohorts (Fig. 2 and Supplementary Fig. 2). The incremental prognostic value of SHR over ABG was further supported by significant improvement in reclassification and discrimination indices (NRI = 0.41, 95% CI 0.25–0.61; IDI = 0.04, 95% CI 0.02–0.08).

Interestingly, the best cut-off identified with Youden’s index for the SHR was 1.14, regardless of the diabetic status. In contrast, for ABG, the best cut-off was 139 mg/dL in the overall population, 252 mg/dL in diabetic patients, and 163 mg/dL in non-diabetic patients (Fig. 2).

Similar ROC findings were observed in the sensitivity analysis excluding patients with extreme ABG and HbA1c values, with SHR showing the highest AUC and confirming 1.14 as the optimal cut-off value, consistent with the overall population (Supplementary Fig. 3).

Therefore, SHR was used to stratify the overall study population into patients with SHR values > 1.14 [high SHR, n = 424 (42.2%)], of whom 258 (60.8%) had DM and those with SHR values ≤ 1.14 [low SHR, n = 581 (57.8%)], of whom 203 (34.9%) were diabetic. The mean SHR of the overall study population was 1.18 ± 0.4; patients with SHR > 1.14 had significantly higher ABG and HbA1c values than those with SHR ≤ 1.14.

### Baseline and procedural characteristics

Baseline characteristics, cardiovascular risk factors, and comorbidities are reported in Table 1. The mean age of the overall study population was 70.3 $\pm$ 12.5 years, and approximately three-quarters were males. Patients with SHR > 1.14 were significantly older, with a higher prevalence of hypertension, DM, atrial fibrillation and chronic kidney disease (*p* < 0.05 for all). The other comorbidities were similar in the two groups, except for peripheral artery disease, which was more frequent among patients with SHR > 1.14 (*p* < 0.001). At hospital admission, patients with higher SHR had higher Killip class and Global Registry of Acute Coronary Events (GRACE) score and a lower rate of typical angina (*p* ≤ 0.006 for all). Furthermore, they exhibited a larger infarct size, as demonstrated by a lower left ventricular ejection fraction (LVEF) and higher peak cTn levels (*p* < 0.001 for both) compared to patients with SHR ≤ 1.14.

**Table 1:** Baseline characteristics of the study population

	Total N = 1005	SHR ≤ 1.14 N = 581	SHR > 1.14 N = 424	*p*-value
Age, years	70.3 ± 12.5	68.8 ± 12.9	72.4 ± 11.5	< 0.001
Female sex, n (%)	256 (25.5)	143 (24.6)	113 (26.7)	0.464
BMI, Kg/m^2^	27.6 ± 4.8	27.4 ± 4.7	27.8 ± 4.9	0.173
*Cardiovascular risk factors*				
Current/past smoking, n (%)	602 (59.9)	357 (61.4)	245 (57.8)	0.242
Hypertension, n (%)	758 (75.4)	423 (72.8)	335 (79.0)	0.024
Dyslipidemia, n (%)	642 (63.9)	377 (64.9)	265 (62.5)	0.436
Diabetes, n (%)	461 (45.9)	203 (34.9)	258 (60.8)	< 0.001
*Medical history*				
Previous MI, n (%)	275 (27.4)	147 (25.3)	128 (30.2)	0.086
Previous PCI, n (%)	261 (26.0)	147 (25.3)	114 (26.9)	0.597
Previous CABG, n (%)	81 (8.1)	45 (7.7)	36 (8.5)	0.668
Previous stroke, n (%)	70 (7.0)	37 (6.4)	33 (7.8)	0.384
PAD, n (%)	107 (10.6)	43 (7.4)	64 (15.1)	< 0.001
CKD, n (%)	322 (32.0)	145 (25.0)	177 (41.7)	< 0.001
Atrial fibrillation, n (%)	111 (11.0)	45 (7.7)	66 (15.6)	< 0.001
*Clinical presentation*				
Angina, n (%)	850 (84.6)	507 (87.3)	343 (80.9)	0.006
Killip class ≥ 2, n (%)	167 (16.6)	60 (10.3)	107 (25.2)	< 0.001
GRACE score > 140, n (%)	488 (48.6)	241 (41.5)	247 (58.3)	< 0.001
LV-EF bp, %	51.7 ± 10.7	53.4 ± 10.1	49.5 ± 11.2	< 0.001
Time symptoms–balloon, hours	30.6 (25.6–50.0)	30.2 (25.5–47.8)	31.2 (25.7–51.3)	0.375
Time hospital admission–balloon, hours	25.3 (20.2–44.9)	25.1 (20.3–42.6)	25.6 (20.1–45.8)	0.874
*Laboratory parameters*				
ABG, mg/dL	160.9 ± 76.5	120.4 ± 33.0	216.4 ± 83.9	< 0.001
HbA1c, mmol/mol	46.0 ± 15.1	44.1 ± 13.2	48.7 ± 17.0	< 0.001
SHR	1.18 ± 0.4	0.93 ± 0.15	1.52 ± 0.39	< 0.001
Haemoglobin, g/dL	13.5 ± 2.0	13.5 ± 2.0	13.4 ± 2.1	0.576
Creatinine, mg/dL	1.22 ± 0.98	1.08 ± 0.55	1.40 ± 1.35	< 0.001
Peak troponin pre-PCI, x URL	40.6 (9.8–160)	31 (7.9–127)	58.4 (11.8–203)	< 0.001
Peak troponin post-PCI, x URL	44.1 (10.5–167)	37.5 (8.6–140)	64.4 (14.1–186)	0.001
*Admission medical therapy*				
SAPT, n (%)	428 (42.6)	246 (42.3)	182 (42.9)	0.853
DAPT, n (%)	74 (7.4)	41 (7.1)	33 (7.8)	0.663
Beta-blockers, n (%)	470 (46.8)	257 (44.2)	213 (50.2)	0.060
RAAS inhibitors, n (%)	576 (57.3)	328 (56.5)	248 (58.5)	0.519
Statins, n (%)	420 (41.8)	232 (39.9)	188 (44.3)	0.162
OHAs, n (%)	302 (30.0)	126 (21.7)	176 (41.5)	< 0.001
Metformin, n (%)	244 (24.3)	102 (17.6)	142 (33.5)	< 0.001
Sulfonylureas, n (%)	90 (9.0)	28 (4.8)	62 (14.6)	< 0.001
DPP-4 Inhibitors, n (%)	33 (3.3)	15 (2.6)	18 (4.2)	0.144
GLP-1 Agonist, n (%)	12 (1.2)	7 (1.2)	5 (1.2)	0.971
SGLT-2 Inhibitors, n (%)	16 (1.6)	12 (2.1)	4 (0.9)	0.160
Insulin, n (%)	129 (12.8)	59 (10.2)	70 (16.5)	0.003

*SHR* Stress hyperglycemia ratio; *BMI* Body mass index; *MI* Myocardial infarction; *PCI* Percutaneous coronary intervention; *CABG* Coronary artery bypass graft; *PAD* Peripheral artery disease; *CKD* Chronic kidney disease; *GRACE* Global Registry of Acute Coronary Events; *LV-EF bp* Left ventricular ejection fraction Simpson biplane evaluated by transthoracic echocardiogram; *ABG* Admission blood glucose; *HbA1c* Glycated hemoglobin; *URL *Upper reference limit; *SAPT* Single antiplatelet therapy; *DAPT* Dual antiplatelet therapy; *RAAS* Renin-angiotensin-aldosterone system; *OHAs* Oral hypoglycemic agents; *DPP-4* Dipeptidylpeptidase 4; *GLP-1* Glucagon-like peptide-1; *SGLT-2* Sodium-glucose cotransporter-2

No differences were found between the two groups regarding cardiovascular therapy at admission. The angiographic and procedural characteristics are shown in Table 2. The median time from symptom onset to PCI did not differ between the two groups. Patients with SHR > 1.14 had a higher rate of multivessel disease (*p* < 0.001), while no differences were observed in the rate of left main-only disease or lesions on venous or arterial coronary grafts. Complex PCI procedures (and all individual components included in the definition) were significantly more frequent in patients with SHR > 1.14 compared to those with SHR ≤ 1.14. In contrast, the latter group showed a higher rate of complete revascularization (*p* < 0.001).

**Table 2:** Description of the angiographic findings and procedural characteristics

	Total N = 1005	SHR ≤ 1.14 N = 581	SHR > 1.14 N = 424	*p*-value
*Angiographic findings*				
Single-vessel disease, n (%)	382 (38.0)	269 (46.3)	113 (26.7)	< 0.001
Multivessel disease (two or more), n (%)	621 (61.8)	311 (53.5)	310 (73.1)	< 0.001
Left main disease only, n (%)	10 (1.0)	5 (0.9)	5 (1.2)	0.615
Lesion on venous or arterial coronary graft, n (%)	60 (6.0)	36 (6.2)	24 (5.7)	0.723
*Percutaneous coronary intervention*				
Complex PCI, n (%)	396 (39.4)	195 (33.6)	201 (47.4)	< 0.001
Multivessel PCI, n (%)	281 (28.0)	136 (23.4)	145 (34.2)	< 0.001
Number of stents implanted ≥ 3, n (%)	210 (20.9)	95 (16.4)	115 (27.1)	< 0.001
Number of lesions treated ≥ 3, n (%)	224 (22.3)	101 (17.4)	123 (29.0)	< 0.001
Bifurcation with stents implanted ≥ 2, n (%)	14 (1.4)	4 (0.7)	10 (2.4)	0.026
Total stent length ≥ 60 mm, n (%)	195 (19.4)	90 (15.5)	105 (24.8)	< 0.001
Chronic total occlusion treated, n (%)	19 (1.9)	11 (1.9)	8 (1.9)	0.994
GpIIb/IIIa inhibitors bail-out use, n (%)	26 (2.6)	14 (2.4)	12 (2.8)	0.678
Final TIMI flow grade < 3, n (%)	96 (9.6)	34 (5.9)	62 (14.6)	< 0.001
Maximum stent diameter, mm	3.17 ± 0.52	3.16 ± 0.53	3.17 ± 0.51	0.984
PCI with DES only, n (%)	905 (90.0)	529 (91.0)	376 (88.7)	0.215
PCI with BMS only, n (%)	23 (2.3)	10 (1.7)	13 (3.1)	0.159
PCI with balloon only, n (%)	51 (5.1)	28 (4.8)	23 (5.4)	0.666
Atherectomy, n (%)	43 (4.3)	22 (3.8)	21 (5.0)	0.367
Complete revascularization, n (%)	592 (58.9)	373 (64.2)	219 (51.7)	< 0.001

*SHR* Stress hyperglycemia ratio; *PCI* Percutaneous coronary intervention; *GpIIb/IIIa* Glycoprotein IIb/IIIa; *TIMI* Thrombolysis in myocardial infarction; *DES* Drug-eluting stent; *BMS* Bare-metal stents

### Impact of glucometabolic status on type 4a MI

**Fig. 3:**
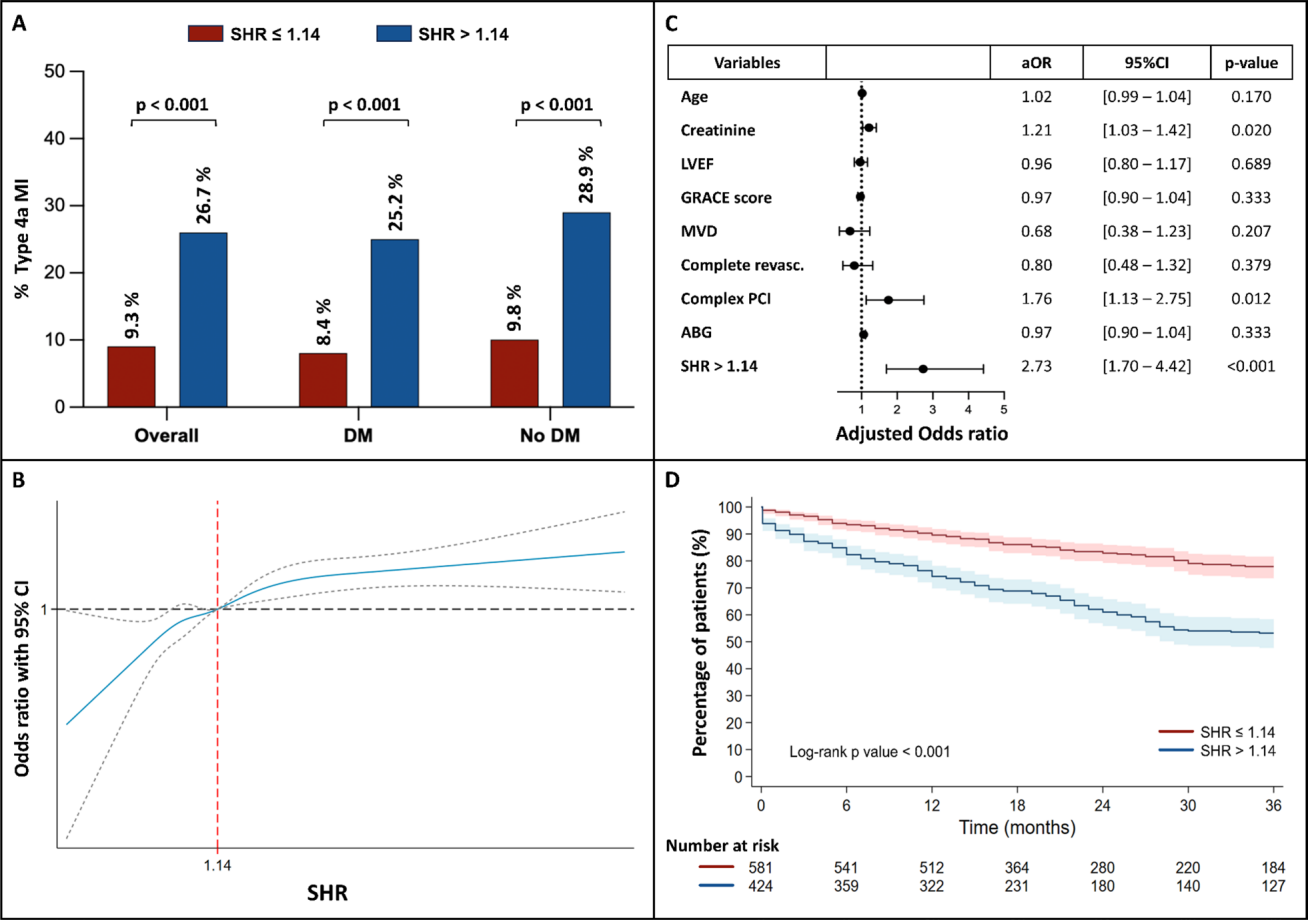
**A** Occurrence of type 4a MI in the overall population, and in diabetic and non-diabetic patients, stratified by the optimal SHR cut-off identified by ROC analysis. **B** Multivariable Cox regression model highlighting the independent predictors of type 4a MI. **C** Restricted cubic spline showing the relationship between SHR values and the incidence of type 4a MI. The black dashed horizontal line represents an odds ratio of 1.0, the blue line indicates the estimated odds ratio, and the shaded grey ribbons represent the 95% confidence interval. **D** Kaplan–Meier curves for MACEs-free survival stratified by the optimal SHR cut-off identified by ROC analysis. *SHR* Stress hyperglycemia ratio; *MI* Myocardial infarction; *DM* Diabetes mellitus; *OR* Odds ratio; *CI* Confidence interval; *LVEF* Left ventricular ejection fraction; *GRACE* Global Registry of Acute Coronary Events; *MVD* Multivessel disease; *PCI* Percutaneous coronary intervention; *ABG* Admission blood glucose; *ROC* Receiver operating characteristic; *MACEs* Major adverse cardiovascular events

Patients with SHR > 1.14 had a threefold higher incidence of type 4a MI compared to those with SHR ≤ 1.14 (26.7% vs. 9.3%, *p* < 0.001) (Fig. 3A and Supplementary Table 2). Figure 3B illustrates a clear upward trend in the incidence of type 4a MI along with increasing SHR values (cut-off of 1.14) whereas Supplementary Fig. 4A shows no such trend for ABG (cut-off of 139 mg/dL).

In the multivariable logistic regression model, after adjusting for confounding factors, high SHR was identified as an independent predictor of type 4a MI occurrence (aOR = 2.73; 95% CI 1.70–4.42; *p* < 0.001), along with complex PCI (aOR = 1.76; 95% CI 1.13–2.75; *p* = 0.012) and higher creatinine levels (aOR = 1.21; 95% CI 1.03–1.42; *p* = 0.002) (Fig. 3C). Notably, neither DM, ABG, nor HbA1c were independent predictors of type 4a MI in the model (Table 3). Consistent results were observed in the sensitivity analysis, where SHR remained independently associated with type 4a MI after exclusion of patients with extreme ABG and HbA1c values (Supplementary Table 3).

**Table 3:** Univariate and multivariable logistic regression model showing the independent predictors of type 4a myocardial infarction in NSTEMI patients

	Unadjusted OR (95% CI)	*p*-value	Adjusted OR (95% CI)	*p*-value
Age	1.02 (1.01–1.04)	0.003	1.02 (0.99–1.04)	0.170
Gender, female	1.10 (0.75–1.59)	0.632	–	–
Hypertension	1.13 (0.77–1.69)	0.549	–	–
Diabetes	1.17 (0.84–1.63)	0.359	–	–
PAD	1.34 (0.80–2.18)	0.248	–	–
Atrial fibrillation	1.12 (0.65–1.83)	0.674	–	–
Creatinine, mg/dL	1.30 (1.13–1.49)	< 0.001	1.21 (1.03–1.42)	0.020
LVEF, %	0.87 (0.75–1.01)	0.074	0.96 (0.80–1.17)	0.689
Peak troponin pre-PCI, X URL	0.95 (0.86–1.04)	0.260	–	–
GRACE score (per 10-unit increase)	1.06 (1.02–1.11)	0.009	0.97 (0.90–1.04)	0.333
Multivessel disease	1.40 (0.99–2.01)	0.060	0.68 (0.38–1.23)	0.207
Complete revascularization	0.72 (0.51–1.00)	0.051	0.80 (0.48–1.32)	0.379
Complex PCI	1.76 (1.26–2.46)	0.001	1.76 (1.13–2.75)	0.012
ABG (per 10-unit increase), mg/dL	1.05 (1.03–1.07)	< 0.001	0.97 (0.90–1.04)	0.333
HbA1c, mmol/mol	1.00 (0.98– 1.01)	0.524	–	–
SHR > 1.14	3.55 (2.50–5.08)	< 0.001	2.73 (1.70–4.42)	< 0.001

Variables associated with type 4a MI in univariate regression (*p*-value < 0.1) were included in the multivariable model

*MI* Myocardial infarction; *NSTEMI* Non–ST-segment elevation myocardial infarction; *OR* Odds ratio; *CI* Confidence interval; *PAD* Peripheral artery disease; *LVEF* Left ventricular ejection fraction evaluated by transthoracic echocardiogram; *PCI* Percutaneous coronary intervention; *URL* Upper reference limit; *GRACE* Global Registry of Acute Coronary Events; *ABG* Admission blood glucose; *HbA1c* Glycated hemoglobin; *SHR* Stress hyperglycemia ratio

In addition, interaction terms between SHR and age, creatinine, and LVEF were tested in the multivariable model, but none showed statistical significance (all *p* > 0.05; Supplementary Table 4).

### Glucometabolic status and clinical outcomes

Overall, 9 patients (0.9%), all with a SHR > 1.14, died during hospitalization due to cardiovascular causes. Patients with SHR > 1.14 had a higher rate of new-onset cardiac arrhythmias, longer hospital stays, and greater use of mechanical circulatory support compared to those with SHR ≤ 1.14 (*p* < 0.014, *p* < 0.001, and *p* = 0.019, respectively– Supplementary Table 2).

Discharge medications were comparable, except for a higher prescription rate of diuretics and oral anticoagulants in patients with SHR > 1.14 (Supplementary Table 5).

The median follow-up duration after discharge was 26 (IQR, 15–52) months. During this period, 178 deaths (17.7%) were recorded, of which 130 (12.9%) were attributed to cardiovascular causes. Additionally, 92 patients (9.2%) experienced recurrent AMI (re-AMI), 96 (9.6%) underwent unplanned revascularization, 121 (12.0%) were hospitalized for HF, and 342 (34.0%) reached the composite outcome. Cardiovascular mortality, re-AMI, unplanned revascularization, and HF hospitalization were all less frequent in patients with SHR ≤ 1.14 than in those with SHR > 1.14 (*p* < 0.001 for all, Supplementary Table 2).

Kaplan–Meier estimates, shown in Fig. 3D, revealed a significantly poorer composite outcome (MACEs) at 36 months in patients with SHR > 1.14 compared to those with SHR ≤ 1.14 (*p* < 0.001).

Similarly, Kaplan–Meier curves in Supplementary Fig. 4B showed that patients with ABG > 139 mg/dL had a significantly higher likelihood of MACEs than those with ABG ≤ 139 mg/dL (*p* < 0.001).

## Discussion

This is the first study to investigate the role of glucometabolic status in predicting type 4a MI in NSTEMI patients undergoing PCI. The main findings were as follows: (i) ABG and SHR were associated with the occurrence of type 4a MI, unlike HbA1c; (ii) SHR emerged as the most accurate glucometabolic parameter for predicting type 4a MI risk, regardless of diabetes status; (iii) the optimal SHR cut-off for predicting type 4a MI was 1.14 in both diabetic and non-diabetic patients; (iv) SHR, unlike ABG and HbA1c, was an independent predictor of type 4a MI, with SHR > 1.14 conferring a threefold increased risk; (v) High SHR was linked to worse in-hospital and long-term outcomes in this population.

### SHR and its association with type 4a MI

It is well established that glucose metabolism is strongly associated with both short- and long-term clinical outcomes in patients with MI, including ST-segment elevation myocardial infarction (STEMI) and NSTEMI [27]. However, no previous study has specifically investigated the association between glucometabolic parameters and the risk of type 4a MI in NSTEMI patients undergoing PCI, nor has it identified which of these parameters provides the strongest predictive value for this complication. This represents a relevant gap in the literature, especially considering that PCI is the routine treatment strategy for NSTEMI patients, and that periprocedural complications such as type 4a MI are not uncommon and carry important prognostic implications [1].

In recent years, the range of glucometabolic markers has expanded to include the SHR, which carries clinical relevance irrespective of diabetes status [28, 29]. In our study, SHR showed the highest predictive performance for type 4a MI, exceeding that of ABG and HbA1c, although ABG showed modest accuracy. Notably, SHR performed well in both diabetic and non-diabetic patients, likely due to its ability to reflect both acute glucose fluctuations and chronic glycemic control, providing broader prognostic value than single-point measures [11]. Notably, the consistency of SHR cut-off at 1.14 across diabetic and non-diabetic cohorts underscores the robustness of this parameter, making it easier to implement as a risk stratification tool in clinical practice. In non-diabetic patients, acute glucose fluctuations may reflect the severity of stress-induced hyperglycemia, which is often a marker of heightened inflammatory and neurohormonal activation [5]. In diabetic patients, SHR incorporates baseline hyperglycemia, which is indicative of chronic metabolic dysregulation, compounding the effects of stress-induced glucose changes during acute illness [11].

From a pathophysiological perspective, the correlation between elevated SHR and the risk of developing type 4a MI may stem from several interrelated mechanisms. Elevated glucose levels have been shown to increase oxidative stress, impair endothelial dysfunction, and promote platelet hyperactivity, all of which can exacerbate vascular injury during PCI [30]. Additionally, stress hyperglycemia amplifies inflammatory signaling, including the upregulation of pro-inflammatory cytokines (e.g., interleukin-6, tumor necrosis factor-alpha) and adhesion molecules (e.g., intercellular adhesion molecule-1, vascular cell adhesion molecule-1), contributing to leukocyte recruitment and vascular inflammation [31, 32]. These processes may promote plaque instability and procedural complications [33]. Furthermore, elevated glucose levels can impair microvascular perfusion by inducing endothelial swelling, glycocalyx degradation, and capillary plugging, mechanisms that have been implicated in the pathogenesis of the no-reflow phenomenon [34, 35]. Finally, stress hyperglycemia has also been significantly associated with larger thrombus burden at coronary angiography, placing these lesions at higher risk of distal embolization or no reflow and inevitably making type 4a MI more frequent [10]. All these mechanisms collectively might explain why SHR, which captures both acute and baseline glycemic status, distinguishing it from ABG and HbA1c in its ability to predict type 4a MI.

Remarkably, the increase in glucose levels observed during the first hours of hospitalization for AMI is a highly common phenomenon [27]. This rise is typically induced by the acute release of catecholamines, cytokines, and cortisol during the early phase of MI [36]. These stress-related hormones trigger a cascade of metabolic responses, including increased hepatic glucose production, insulin resistance, and impaired glucose uptake by peripheral tissues, rendering the entire cardiovascular system more vulnerable to inflammatory and hypoxic injury during this critical period and potentially impacting prognosis [37]. In line with these pathophysiological mechanisms, we found that, beyond predicting type 4a MI, elevated SHR and ABG were also associated with poor long-term prognosis in patients with NSTEMI, consistent with findings from previous studies [12, 38–40].

The identification of SHR as an independent factor associated with both type 4a MI and long-term MACEs in NSTEMI patients has significant clinical implications. First, SHR calculation is simple and feasible for routine use in clinical practice as it only requires ABG and HbA1c values, parameters readily available in most healthcare settings. Moreover, the identified risk threshold of 1.14 remains consistent across both diabetic and non-diabetic patients, further supporting its applicability in diverse clinical populations. Second, incorporating SHR into risk stratification models could enhance the identification of high-risk patients, allowing for targeted measures during PCI such as heightened awareness of periprocedural risk, careful planning to minimize ischemic time, use of intravascular imaging, stricter glycemic control, more vigilant post-procedure monitoring or escalation of therapy. Notably, widely recognized prognostic tools such as the GRACE score and Killip classification, while well-established for risk stratification in NSTEMI, are not designed to specifically predict the risk of type 4a MI. Therefore, incorporating SHR into existing models might enhance their prognostic accuracy and clinical utility. Finally, the association between high SHR and adverse long-term outcomes, including MACEs, underscores the need to address glucometabolic dysregulation as part of comprehensive secondary prevention strategies in AMI patients. Although SHR is simple to calculate, its use in routine care may be limited by the availability of HbA1c at the time of admission. Nonetheless, as early metabolic profiling, including HbA1c, is increasingly incorporated into acute coronary syndrome protocols, the clinical applicability of SHR is likely to expand.

### Study limitations

This study has some limitations that should be acknowledged. First, this is an observational study and, although we adjusted for key confounders in the regression analyses, residual confounding cannot be excluded. Additionally, ABG levels may have been influenced by various factors such as the composition and timing of the last meal, as well as the time of day when measurements were taken. Moreover, the lack of an external validation cohort limits the generalizability and reproducibility of our findings. Finally, while SHR demonstrated prognostic value, its role in guiding therapeutic interventions based on acute glycemic status has not been prospectively validated. Therefore, our findings should be considered exploratory, and further studies are required to validate the prognostic impact of SHR in predicting type 4a MI and long-term outcomes in patients with NSTEMI undergoing PCI.

## Conclusions

SHR has emerged as a robust glucometabolic parameter with significant prognostic value in patients with NSTEMI undergoing PCI. Its ability to integrate acute glucose fluctuations and baseline glycemic control provides a superior predictive tool for type 4a MI compared to traditional snapshot measures such as ABG and HbA1c. The findings of this study underscore the potential of SHR as a simple and practical risk stratification tool that can help identify patients at higher risk of developing type 4a MI, potentially allowing for tailored therapeutic interventions aimed at reducing periprocedural ischemic events and for more intensive follow-up strategies.

## Supplementary Information

Supplementary Material 1.

## Data Availability

No datasets were generated or analysed during the current study.

## Abbreviations

ABG: Admission blood glucose
AMI: Acute myocardial infarction
cTn: Cardiac troponin
DM: Diabetes mellitus
HbA1c: Glycated hemoglobin
HF: Heart failure
ICA: Invasive coronary angiography
LVEF: Left ventricular ejection fraction
MACEs: Major adverse cardiovascular events
SHR: Stress hyperglycemia ratio
UDMI: Universal definition of myocardial infarction
