# Supplementary material for: Impact of glucometabolic status on type 4a myocardial infarction in patients with non–ST-segment elevation myocardial infarction: the role of stress hyperglycemia ratio
Source: Cardiovasc Diabetol. 2025 Oct 11;24:394. doi: 10.1186/s12933-025-02837-y (PMC12514836; doi:10.1186/s12933-025-02837-y)
Supplement: Supplementary file 1 — Supplementary Material 1. [file 12933_2025_2837_MOESM1_ESM.docx]

**Impact of Glucometabolic Status on Type 4a Myocardial Infarction in Patients With**

**Non–ST-Segment–Elevation Myocardial Infarction: The Role of Stress Hyperglycemia Ratio**

**Authors**: Matteo Armillotta, MD † ^a,b^; Luca Bergamaschi, MD † ^a,b^; Francesco Angeli, MD ^a,b^; Marta Belmonte, MD ^c,d^; Marcello Casuso Alvarez, MD ^a,e^; Angelo Sansonetti, MD ^a,e^; Damiano Fedele, MD ^a,e^; Sara Amicone, MD ^a,b^; Lisa Canton, MD ^a,b^; Davide Bertolini, MD ^a,e^; Andrea Impellizzeri, MD ^a,e^; Francesca Bodega, MD ^a,e^; Nicole Suma, MD ^a,e^; Francesco Pio Tattilo, MD ^a,e^; Daniele Cavallo, MD ^a,e^; Ornella Di Iuorio, MD ^a,e^; Khrystyna Ryabenko, MD ^a,e^; Andrea Rinaldi, MD, PhD ^e^; Francesco Saia, MD, PhD ^a,e^; Gianni Casella, MD ^f^; Jacopo Lenzi, PhD ^g^; Paola Rucci, PhD ^g^; Pasquale Paolisso, MD, PhD * ^d^; Carmine Pizzi, MD * ^a,b^.

**Affiliations:**

1. Department of Medical and Surgical Sciences – DIMEC – Alma Mater Studiorum, University of Bologna, Bologna, Italy
2. Cardiovascular Division, Morgagni-Pierantoni University Hospital, Forlì, Italy
3. Department of Advanced Biomedical Sciences, University Federico II, Naples, Italy
4. Cardiology Unit, Sant'Andrea University Hospital, Rome, Italy
5. Cardiology Unit, IRCCS Azienda Ospedaliero-Universitaria di Bologna, Bologna, Italy
6. Unit of Cardiology, Maggiore Hospital, Bologna, Italy
7. Division of Hygiene and Biostatistics, Department of Biomedical and Neuromotor Sciences, Alma Mater Studiorum, University of Bologna, Bologna, Italy

† The first two authors contributed equally to this work

* The last two authors contributed equally to this work

**Corresponding author**

Carmine Pizzi, MD, FESC; Department of Medical and Surgical Sciences (DIMEC), Alma Mater Studiorum - University of Bologna, Bologna, Italy; Cardiovascular Division, Morgagni-Pierantoni University Hospital, Via Carlo Forlanini 34, 47121, Forlì, Italy. Tel. +39 0543 738148; Fax +39 0543 738636. E-mail: [carmine.pizzi@unibo.it](mailto:carmine.pizzi@unibo.it)

**SUPPLEMENTARY MATERIAL**

**EXTENDED METHODS**

**Data Collection**

For each patient, demographic and baseline clinical data were prospectively collected, including age, sex, anthropometric data, cardiovascular risk factors, family history of cardiovascular disease, history of comorbidities, admission and discharge medical therapy. Upon admission, blood samples were collected for routine laboratory analyses, including complete blood count, electrolytes, creatinine, lipid profile, glucose, and cardiac troponin (cTn). The glomerular filtration rate (GFR) was calculated using the CKD-EPI formula. Chronic kidney disease was defined as kidney damage or GFR <60 mL/min/1.73 m^2^ for 3 months or more, irrespective of cause (1).

Cardiac troponin levels were measured at hospital admission (0 h), every 3 hours until the peak level was identified, and within 1 hour prior to coronary angiography. Following percutaneous coronary intervention (PCI), at least three cTn measurements were obtained: immediately after the procedure, and at 3 and 6 hours post-PCI. If post-PCI cTn levels increased or if clinically indicated (e.g., new ischemic symptoms or ECG changes), additional measurements were performed every 3 hours to monitor peak post-PCI levels within the first 48 hours (2).

All patients underwent a standard 12-lead ECG at the time of initial medical contact, upon arrival at the cardiac intensive care unit (CICU), prior to PCI, within 1 hour post-PCI (upon returning to the CICU), and every morning until discharge. Additionally, all patients received at least one 2D transthoracic echocardiography (TTE) at the time of non-ST-elevation myocardial infarction (NSTEMI) diagnosis and another within 48 hours post-PCI (2). Further ECG and TTE evaluations were conducted as clinically indicated.

2D TTE was performed according to the latest European guidelines by experienced operators (3). At least 3 consecutive beats were recorded for each view, and all images were stored for offline analysis. Left ventricular ejection fraction (LVEF) was calculated with the biplane Simpson’s method according to the European Association of Cardiovascular Imaging guidelines (4).

Coronary angiography and PCI were performed during hospitalization by expert interventional cardiologists in accordance with European guidelines (5-7). Detailed procedural information were systematically collected. Complex PCI was defined by the presence of at least one of the following criteria: multivessel stenting, implantation of at least three stents, treatment of at least three lesions, bifurcation requiring two stents, total stent length exceeding 60 mm, or PCI of a chronic total occlusion (8-10). Complete revascularization was defined as PCI performed on all vessels suitable for successful treatment, with a diameter of at least 2.5 mm and a stenosis of ≥70% by visual estimation (or 50–69% by visual estimation with FFR ≤0.80), in the absence of a graft (11).

**Clinical Outcomes Definitions**

Major adverse cardiovascular events (MACEs) were defined as the occurrence of the first among the following events:

- **All-cause mortality**: Death from any cause occurring during the follow-up period.
- **Reinfarction**: Diagnosed as acute myocardial injury with clinical evidence of acute myocardial ischemia occurring after 48 hours from PCI during the follow-up. This required a rise and/or fall in cTn values, with at least one value above the 99^th^ percentile URL, along with at least one of the following criteria:
  - Symptoms of myocardial ischemia
  - New ischemic ECG changes
  - Development of pathological Q waves
  - Imaging evidence of new loss of viable myocardium or new regional wall motion abnormality consistent with an ischemic etiology
  - Identification of a coronary thrombus by angiography or autopsy (12).
- **Unplanned revascularization**: Defined as unanticipated revascularization by either PCI or CABG not planned during the index procedure (13).
- **Stroke**: Defined as an ischemic cerebral infarction caused by embolic or thrombotic occlusion of a major intracranial artery, resulting in a new focal neurologic deficit lasting more than 24 hours (14).
- **Hospitalization for heart failure** (**HF**): Defined as an event meeting the following criteria:
  - Hospitalization: Admission to an inpatient unit or emergency department visit resulting in at least 6 hours of hospitalization
    AND
  - Clinical symptoms of HF: At least one of the following new or worsening symptoms:
    - Dyspnea
    - Orthopnea
    - Paroxysmal nocturnal dyspnea
    - Increasing fatigue or worsening exercise tolerance
      AND
  - Physical signs of HF: At least two of the following:
    - Peripheral edema
    - Pulmonary rales
    - Jugular venous distension
    - Tachypnea (respiratory rate > 20 breaths/minute)
    - Rapid weight gain
    - S3 gallop
    - Increasing abdominal distension or ascites
    - Hepatojugular reflux
    - Radiological evidence of worsening HF
    - Right A right heart catheterization within 24 hours of admission showing a pulmonary capillary wedge pressure (pulmonary artery occlusion pressure) ≥ 18 mm Hg and/or a cardiac output < 2.2 L/min/m2 (15).

Cardiovascular mortality consisted of deaths that result from an acute myocardial infarction (AMI), sudden cardiac death, heart failure, stroke, and other cardiovascular causes (14).

**REFERENCES**

1. Levey AS, Eckardt KU, Tsukamoto Y, Levin A, Coresh J, Rossert J, et al. Definition and classification of chronic kidney disease: a position statement from Kidney Disease: Improving Global Outcomes (KDIGO). Kidney Int. 2005;67(6):2089-100.

2. Armillotta M, Bergamaschi L, Paolisso P, Belmonte M, Angeli F, Sansonetti A, et al. Prognostic Relevance of Type 4a Myocardial Infarction and Periprocedural Myocardial Injury in Patients With Non-ST-Segment-Elevation Myocardial Infarction. Circulation. 2025;151(11):760-72.

3. Recommendations for Cardiac Chamber Quantification by Echocardiography in Adults: An Update from the American Society of Echocardiography and the European Association of, Cardiovascular Imaging. Eur Heart J Cardiovasc Imaging. 2016;17(4):412.

4. Lang RM, Badano LP, Mor-Avi V, Afilalo J, Armstrong A, Ernande L, et al. Recommendations for cardiac chamber quantification by echocardiography in adults: an update from the American Society of Echocardiography and the European Association of Cardiovascular Imaging. Eur Heart J Cardiovasc Imaging. 2015;16(3):233-70.

5. Collet JP, Thiele H, Barbato E, Barthelemy O, Bauersachs J, Bhatt DL, et al. 2020 ESC Guidelines for the management of acute coronary syndromes in patients presenting without persistent ST-segment elevation. Eur Heart J. 2021;42(14):1289-367.

6. Byrne RA, Rossello X, Coughlan JJ, Barbato E, Berry C, Chieffo A, et al. 2023 ESC Guidelines for the management of acute coronary syndromes. Eur Heart J. 2023.

7. Roffi M, Patrono C, Collet JP, Mueller C, Valgimigli M, Andreotti F, et al. 2015 ESC Guidelines for the management of acute coronary syndromes in patients presenting without persistent ST-segment elevation: Task Force for the Management of Acute Coronary Syndromes in Patients Presenting without Persistent ST-Segment Elevation of the European Society of Cardiology (ESC). Eur Heart J. 2016;37(3):267-315.

8. Giustino G, Chieffo A, Palmerini T, Valgimigli M, Feres F, Abizaid A, et al. Efficacy and Safety of Dual Antiplatelet Therapy After Complex PCI. J Am Coll Cardiol. 2016;68(17):1851-64.

9. Valgimigli M, Bueno H, Byrne RA, Collet JP, Costa F, Jeppsson A, et al. 2017 ESC focused update on dual antiplatelet therapy in coronary artery disease developed in collaboration with EACTS: The Task Force for dual antiplatelet therapy in coronary artery disease of the European Society of Cardiology (ESC) and of the European Association for Cardio-Thoracic Surgery (EACTS). Eur Heart J. 2018;39(3):213-60.

10. Mohamed MO, Polad J, Hildick-Smith D, Bizeau O, Baisebenov RK, Roffi M, et al. Impact of coronary lesion complexity in percutaneous coronary intervention: one-year outcomes from the large, multicentre e-Ultimaster registry. EuroIntervention. 2020;16(7):603-12.

11. Mehta SR, Wood DA, Storey RF, Mehran R, Bainey KR, Nguyen H, et al. Complete Revascularization with Multivessel PCI for Myocardial Infarction. N Engl J Med. 2019;381(15):1411-21.

12. Thygesen K, Alpert JS, Jaffe AS, Chaitman BR, Bax JJ, Morrow DA, et al. Fourth Universal Definition of Myocardial Infarction (2018). Circulation. 2018;138(20):e618-e51.

13. De Bruyne B, Pijls NH, Kalesan B, Barbato E, Tonino PA, Piroth Z, et al. Fractional flow reserve-guided PCI versus medical therapy in stable coronary disease. N Engl J Med. 2012;367(11):991-1001.

14. Hicks KA, Mahaffey KW, Mehran R, Nissen SE, Wiviott SD, Dunn B, et al. 2017 Cardiovascular and Stroke Endpoint Definitions for Clinical Trials. Circulation. 2018;137(9):961-72.

15. McDonagh TA, Metra M, Adamo M, Gardner RS, Baumbach A, Bohm M, et al. 2021 ESC Guidelines for the diagnosis and treatment of acute and chronic heart failure. Eur Heart J. 2021;42(36):3599-726.

**Supplementary Table 1. Baseline characteristics of the final study population compared with patients excluded due to missing HbA1c measurements.**

|  | Total  N = 1732 | Final study population  N = 1005 | Patients without HbA1c data  N = 727 | *p-value* |
| --- | --- | --- | --- | --- |
| Age, years | 70.7 ± 12.5 | 70.3 ± 12.5 | 71.3 ± 12.6 | *0.086* |
| Female sex, n (%) | 471 (27.2) | 256 (25.5) | 215 (29.6) | *0.058* |
| BMI, Kg/m^2^ | 27.4 ± 7.9 | 27.6 ± 4.8 | 27.1 ± 11.2 | *0.271* |
| Cardiovascular risk factors | | | | |
| Current/past smoking, n (%) | 1045 (60.3) | 602 (59.9) | 443 (60.9) | *0.664* |
| Hypertension, n (%) | 1319 (76.2) | 758 (75.4) | 561 (77.2) | *0.401* |
| Dyslipidemia, n (%) | 1136 (65.6) | 642 (63.9) | 494 (68.0) | *0.078* |
| Diabetes, n (%) | 489 (42.5) | 461 (45.9) | 28 (19.2) | *<0.001* |
| Medical history | | | | |
| Previous MI, n (%) | 472 (27.3) | 275 (27.4) | 197 (27.1) | *0.903* |
| Previous PCI, n (%) | 452 (26.1) | 261 (26.0) | 191 (26.3) | *0.689* |
| Previous CABG, n (%) | 125 (7.2) | 81 (8.1) | 44 (6.1) | *0.111* |
| Previous stroke, n (%) | 117 (6.8) | 70 (7.0) | 47 (6.5) | *0.682* |
| PAD, n (%) | 167 (9.6) | 107 (10.6) | 60 (8.3) | *0.096* |
| CKD, n (%) | 552 (31.9) | 322 (32.0) | 230 (31.6) | *0.859* |
| Atrial fibrillation, n (%) | 200 (11.5) | 111 (11.0) | 89 (12.2) | *0.442* |
| Clinical presentation | | | | |
| Angina, n (%) | 1472 (85.0) | 850 (84.6) | 622 (85.6) | *0.573* |
| Killip class ≥2, n (%) | 187 (16.2) | 167 (16.6) | 20 (13.7) | *0.372* |
| GRACE score >140, n (%) | 851 (49.1) | 488 (48.6) | 363 (49.9) | *0.572* |
| LV-EF bp, % | 52.1 ± 11.0 | 51.7 ± 10.7 | 52.6 ± 11.2 | *0.132* |
| Time symptoms–balloon, hours | 30.5 (25.1-50.8) | 30.6 (25.6-50.0) | 30.4 (24.1-51.3) | *0.826* |
| Laboratory parameters | | | | |
| Haemoglobin, g/dL | 13.5 ± 2.1 | 13.5 ± 2.0 | 13.6 ± 2.3 | *0.396* |
| Creatinine, mg/dL | 1.21 ± 1.0 | 1.22 ± 0.98 | 1.20 ± 1.02 | *0.705* |
| Peak troponin pre-PCI, x URL | 40.3 (10.1-150) | 40.6 (9.8-160) | 39 (10.2-132) | *0.779* |
| Peak troponin post-PCI, x URL | 43.8 (10.7-164) | 44.1 (10.5-167) | 43.8 (10.9-162) | *0.656* |
| Admission medical therapy | | | | |
| SAPT, n (%) | 732 (32.3) | 428 (42.6) | 304 (41.8) | *0.748* |
| DAPT, n (%) | 125 (7.2) | 74 (7.4) | 51 (7.0) | *0.782* |
| Beta-blockers, n (%) | 802 (46.3) | 470 (46.8) | 332 (45.7) | *0.651* |
| RAAS inhibitors, n (%) | 971 (56.1) | 576 (57.3) | 395 (54.3) | *0.217* |
| Statins, n (%) | 691 (39.9) | 420 (41.8) | 271 (37.3) | *0.058* |
| OHAs, n (%) | 359 (20.7) | 302 (30.0) | 57 (7.8) | *<0.001* |
| Metformin, n (%) | 287 (16.6) | 244 (24.3) | 43 (5.9) | *<0.001* |
| Sulfonylureas, n (%) | 115 (6.6) | 90 (9.0) | 25 (3.4) | *<0.001* |
| DPP-4 Inhibitors, n (%) | 40 (2.3) | 33 (3.3) | 7 (1.0) | *0.003* |
| GLP-1 Agonist, n (%) | 14 (0.8) | 12 (1.2) | 2 (0.3) | *0.055* |
| SGLT-2 Inhibitors, n (%) | 19 (1.1) | 16 (1.6) | 3 (0.4) | *0.034* |
| Insulin, n (%) | 166 (9.6) | 129 (12.8) | 37 (5.1) | *<0.001* |

Abbreviations: HbA1c = glycated hemoglobin; BMI = body mass index; MI = myocardial infarction; PCI = percutaneous coronary intervention; CABG = coronary artery bypass graft; PAD = peripheral artery disease; CKD = chronic kidney disease; GRACE = Global Registry of Acute Coronary Events; LV-EF bp = left ventricular ejection fraction Simpson biplane evaluated by transthoracic echocardiogram; ABG = admission blood glucose.

**Supplementary Table 2. Incidence of type 4a myocardial infarction, in-hospital and long-term clinical outcomes.**

|  | Total  N = 1005 | SHR ≤1.14  N = 581 | SHR >1.14  N = 424 | *p-value* |
| --- | --- | --- | --- | --- |
| Type 4a MI, n (%) | 167 (16.6) | 54 (9.3) | 113 (26.7) | *<0.001* |
| In-hospital clinical outcomes | | | | |
| In-hospital death, n (%) | 10 (1.0) | 1 (0.2) | 9 (2.1) | *0.002* |
| In-hospital cardiovascular death, n (%) | 9 (0.9) | 0 (0.0) | 9 (2.1) | *<0.001* |
| Mechanical circulatory support, n (%) | 17 (1.7) | 6 (1.0) | 13 (3.1) | *0.019* |
| IABP, n (%) | 12 (1.2) | 4 (0.7) | 8 (1.9) | *-* |
| Impella, n (%) | 6 (0.6) | 2 (0.3) | 4 (0.9) | *-* |
| ECMO, n (%) | 4 (0.4) | 1 (0.2) | 3 (0.7) | *-* |
| In-hospital arrhythmias, n (%) | 53 (5.3) | 22 (3.8) | 31 (7.3) | *0.014* |
| Length of hospital stay, days | 5 (3-7) | 4 (3-6) | 5 (4-9) | *<0.001* |
| Long-term clinical outcomes | | | | |
| MACEs, n (%) | 342 (34.0) | 128 (22.0) | 214 (50.5) | *<0.001* |
| All-cause death, n (%) | 178 (17.7%) | 61 (10.5) | 117 (27.6) | *<0.001* |
| Cardiovascular death, n (%) | 130 (12.9) | 42 (7.2) | 88 (20.8) | *<0.001* |
| Re-infarction, n (%) | 92 (9.2) | 31 (5.3) | 61 (14.4) | *<0.001* |
| Unplanned revascularization, n (%) | 96 (9.6) | 38 (6.5) | 58 (13.7) | *<0.001* |
| Stroke, n (%) | 22 (2.2) | 10 (1.7) | 12 (2.8) | *0.235* |
| HF hospitalization, n (%) | 121 (12.0) | 46 (7.9) | 75 (17.7) | *<0.001* |

Abbreviations: IABP = intra-aortic balloon pump; ECM = extracorporeal membrane oxygenation; MI = myocardial infarction; HF = heart failure; MACEs = major adverse cardiovascular events.

**Supplementary Table 3.** **Sensitivity analysis: univariate and multivariable logistic regression model showing independent predictors of type 4a myocardial infarction after exclusion of patients with extreme ABG and HbA1c values.**

|  | Unadjusted OR  (95% CI) | *p-value* | Adjusted OR  (95% CI) | *p-value* |
| --- | --- | --- | --- | --- |
| Age | 1.02 (1.01-1.04) | *0.009* | 1.01 (0.99-1.04) | *0.372* |
| Gender, female | 0.96 (0.58-1.53) | *0.858* | - | *-* |
| Hypertension | 1.48 (0.88-2.61) | *0.158* | - | *-* |
| Diabetes | 1.26 (0.83-1.91) | *0.276* | - | *-* |
| PAD | 1.14 (0.56-2.12) | *0.704* | - | *-* |
| Atrial fibrillation | 1.28 (0.67-2.32) | *0.430* | - | *-* |
| Creatinine, mg/dL | 1.29 (1.11-1.51) | *0.001* | 1.18 (0.98-1.43) | *0.075* |
| LVEF, % | 1.00 (0.98-1.02) | *0.696* | - | *-* |
| Peak troponin pre-PCI, X URL | 0.91 (0.80-1.02) | *0.101* | - | *-* |
| GRACE score (per 10-unit increase) | 1.07 (1.01-1.14) | *0.022* | 0.99 (0.90-1.08) | *0.811* |
| Multivessel disease | 1.22 (0.80-1.89) | *0.354* | - | *-* |
| Complete revascularization | 0.65 (0.43-0.98) | *0.041* | 0.78 (0.47-1.29) | *0.329* |
| Complex PCI | 1.46 (0.96-2.23) | *0.075* | 1.15 (0.70-1.87) | *0.575* |
| ABG (per 10-unit increase), mg/dL | 1.10 (1.05-1.15) | *<0.001* | 1.01 (0.93-1.08) | *0.893* |
| HbA1c, mmol/mol | 0.98 (0.95-1.01) | *0.140* | - | *-* |
| SHR >1.14 | 3.16 (2.07-4.91) | *<0.001* | 2.78 (1.42-5.45) | *0.003* |

*Variables associated with type 4a MI in univariate regression (p-value <0.1) were included in the multivariable model.*

Abbreviations: MI = myocardial infarction; NSTEMI = non-ST-segment elevation myocardial infarction; PAD = peripheral artery disease; LVEF = left ventricular ejection fraction evaluated by transthoracic echocardiogram; PCI = percutaneous coronary intervention; URL = upper reference limit; GRACE = Global Registry of Acute Coronary Events; ABG = admission blood glucose; HbA1c = glycated hemoglobin; SHR = stress hyperglycemia ratio.

**Supplementary Table 4. Interaction terms between SHR and selected clinical variables in the multivariable logistic regression model for predicting type 4a myocardial infarction.**

| Interaction term | Coefficient (β) | Standard Error | Odds Ratio | 95% CI | *p-value* |
| --- | --- | --- | --- | --- | --- |
| SHR × Age | -0.018 | 0.016 | 0.98 | 0.95-1.01 | *0.286* |
| SHR × Creatinine | -0.251 | 0.154 | 0.78 | 0.57-1.05 | *0.104* |
| SHR × LVEF | 0.012 | 0.017 | 1.01 | 0.98-1.05 | *0.486* |

Abbreviations: SHR = stress hyperglycemia ratio; CI = confidence interval; LVEF = left ventricular ejection fraction.

**Supplementary Table 5. Medical therapy prescribed at discharge for patients discharged alive.**

|  | Total  N = 995 | SHR ≤1.14  N = 581 | SHR >1.14  N = 424 | *p-value* |
| --- | --- | --- | --- | --- |
| Discharge medical therapy | | | | |
| Aspirin, n (%) | 976 (98.1) | 569 (98.1) | 407 (98.1) | *0.972* |
| P2Y12 Inhibitor, n (%) | 986 (99.1) | 576 (99.3) | 410 (98.8) | *0.397* |
| DAPT, n (%) | 968 (97.3) | 565 (97.4) | 403 (97.1) | *0.770* |
| Beta-blockers, n (%) | 835 (83.9) | 485 (83.6) | 350 (84.3) | *0.762* |
| RAAS inhibitors, n (%) | 851 (85.5) | 499 (86.0) | 352 (84.8) | *0.591* |
| Diuretics, n (%) | 390 (39.2) | 188 (32.4) | 202 (48.7) | *<0.001* |
| Statins, n (%) | 950 (95.5) | 558 (96.2) | 392 (94.5) | *0.190* |
| Oral anticoagulant, n (%) | 126 (12.7) | 53 (9.1) | 73 (17.6) | *<0.001* |
| OHAs, n (%) | 301 (30.3) | 126 (21.7) | 175 (42.2) | *<0.001* |
| Metformin, n (%) | 228 (22.9) | 101 (17.4) | 127 (30.6) | *<0.001* |
| Sulfonylureas, n (%) | 75 (7.5) | 27 (4.7) | 48 (11.6) | *<0.001* |
| DPP-4 Inhibitors, n (%) | 46 (4.6) | 17 (2.9) | 29 (7.0) | *0.003* |
| GLP-1 Agonist, n (%) | 19 (1.9) | 10 (1.7) | 9 (2.2) | *0.613* |
| SGLT-2 Inhibitors, n (%) | 37 (3.7) | 22 (3.8) | 15 (3.6) | *0.883* |
| Insulin, n (%) | 161 (16.2) | 66 (11.4) | 95 (22.9) | *<0.001* |

Abbreviations: SHR = stress hyperglycemia ratio; DAPT = dual antiplatelet therapy; RAAS = renin-angiotensin-aldosterone system; OHAs = oral hypoglycemic agents; DPP-4 = dipeptidylpeptidase 4; GLP-1 = glucagon-like peptide-1; SGLT-2 = sodium-glucose cotransporter-2.

**SUPPLEMENTARY FIGURES**

**Supplementary Figure 1. Study flowchart.**

**
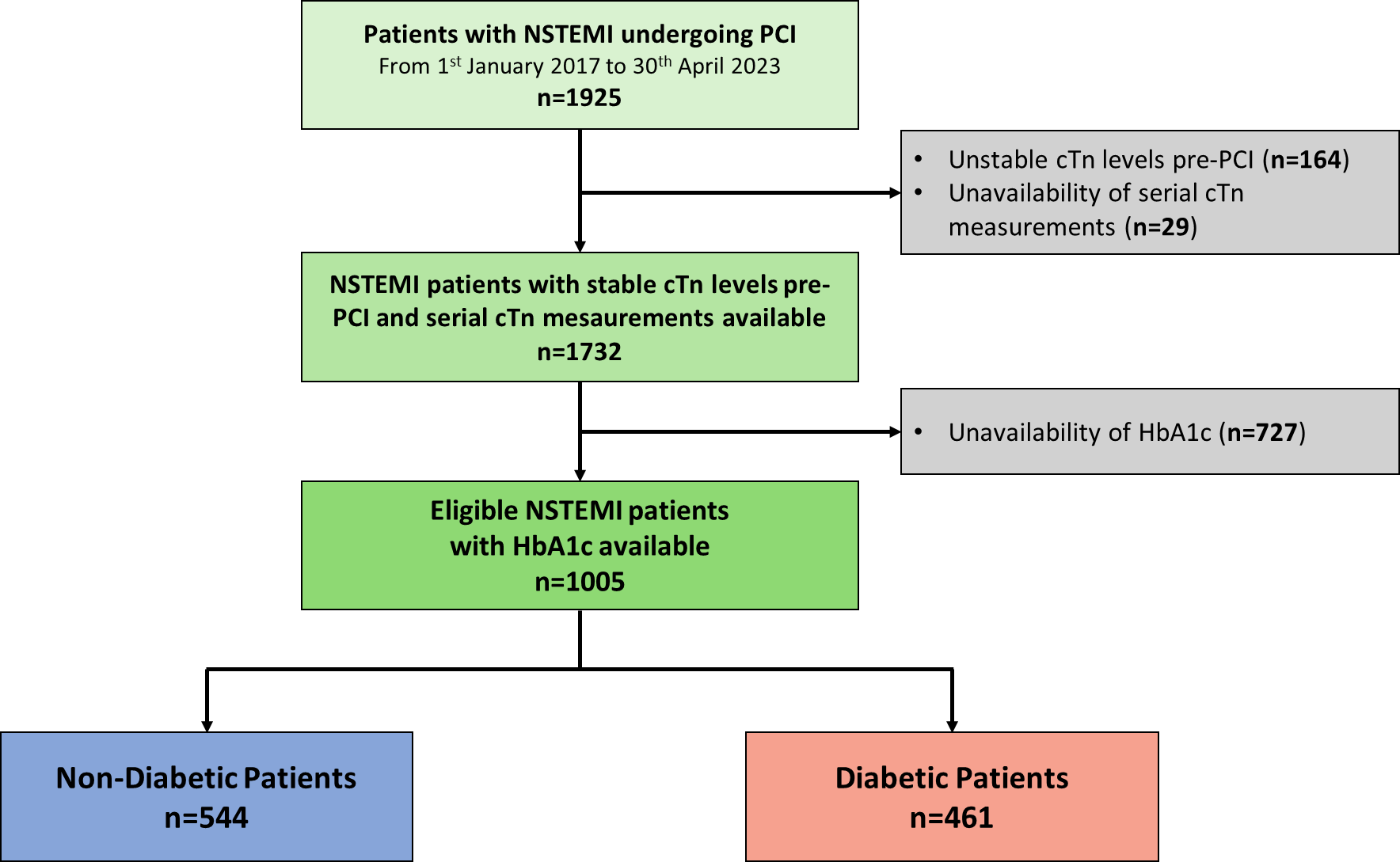
**

Abbreviations: NSTEMI = non-ST-elevation myocardial infarction; PCI = percutaneous coronary intervention; cTn = cardiac troponin; HbA1c = glycosylated hemoglobin.

**Supplementary Figure 2.** **ROC curves for predicting type 4a MI using various glucometabolic parameters in the overall study population, diabetic and non-diabetic patients.**

**
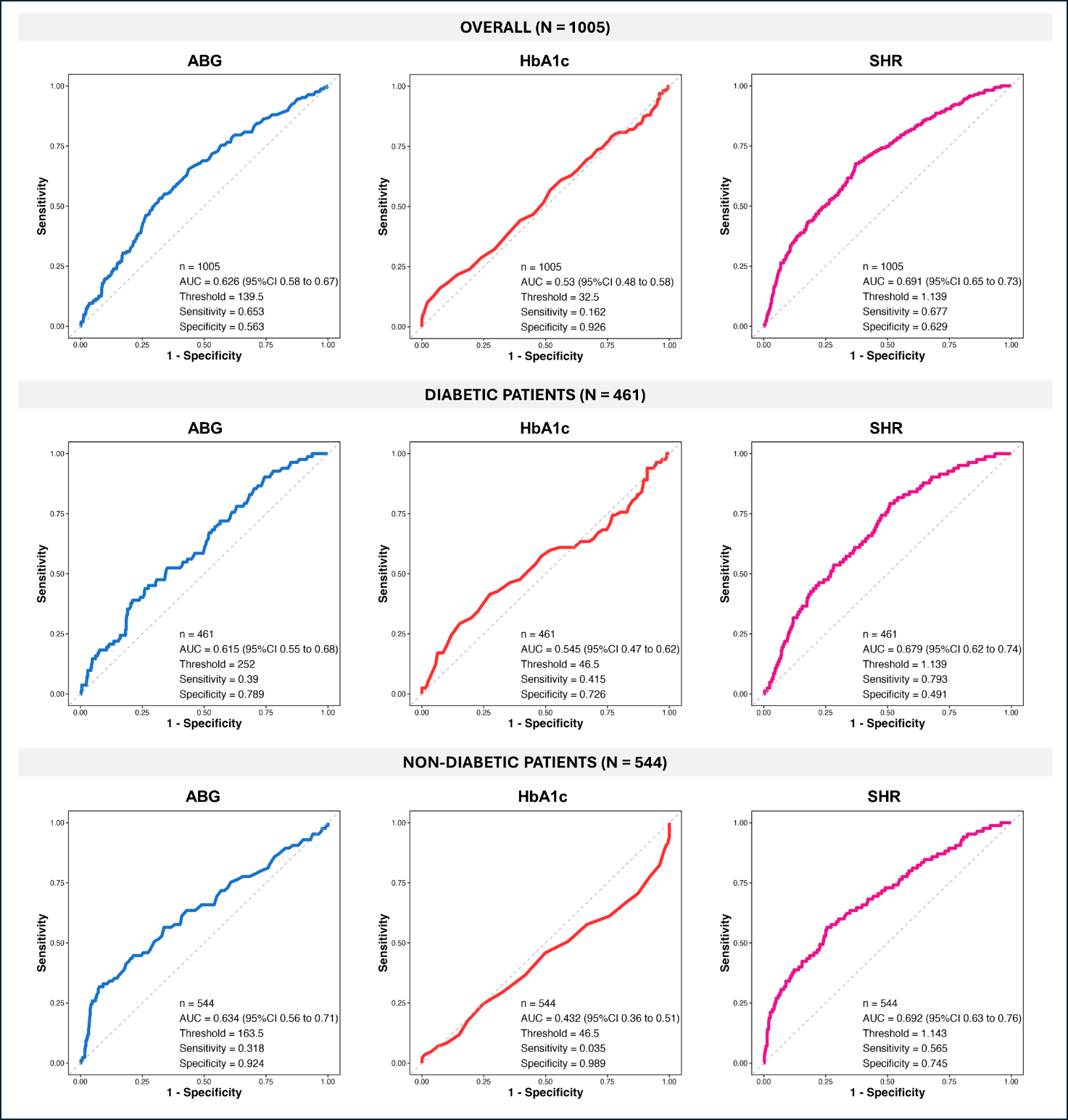
**

Abbreviations: ROC = receiver operating characteristic; MI = myocardial infarction; AUC = area under the curve; ABG = admission blood glucose; HbA1c = glycosylated hemoglobin; SHR = stress hyperglycemia ratio.

**Supplementary Figure 3.** **Sensitivity analysis: ROC curves for predicting type 4a MI using various glucometabolic parameters after exclusion of patients with extreme ABG and HbA1c values.**

**
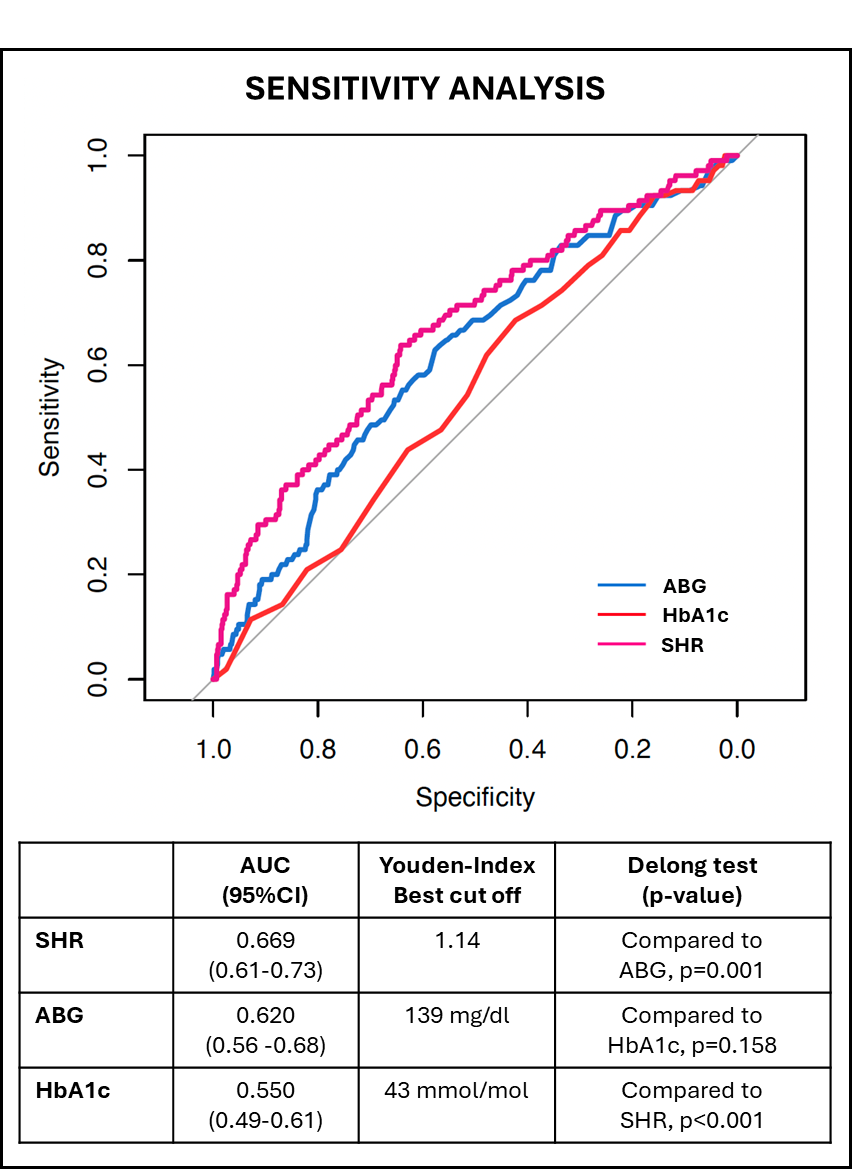
**

Abbreviations: ROC = receiver operating characteristic; MI = myocardial infarction; AUC = area under the curve; ABG = admission blood glucose; HbA1c = glycosylated hemoglobin; SHR = stress hyperglycemia ratio.

**Supplementary Figure 4. Kaplan–Meier curves for MACEs-free survival stratified by the optimal ABG cutoff identified by ROC analysis.**

**
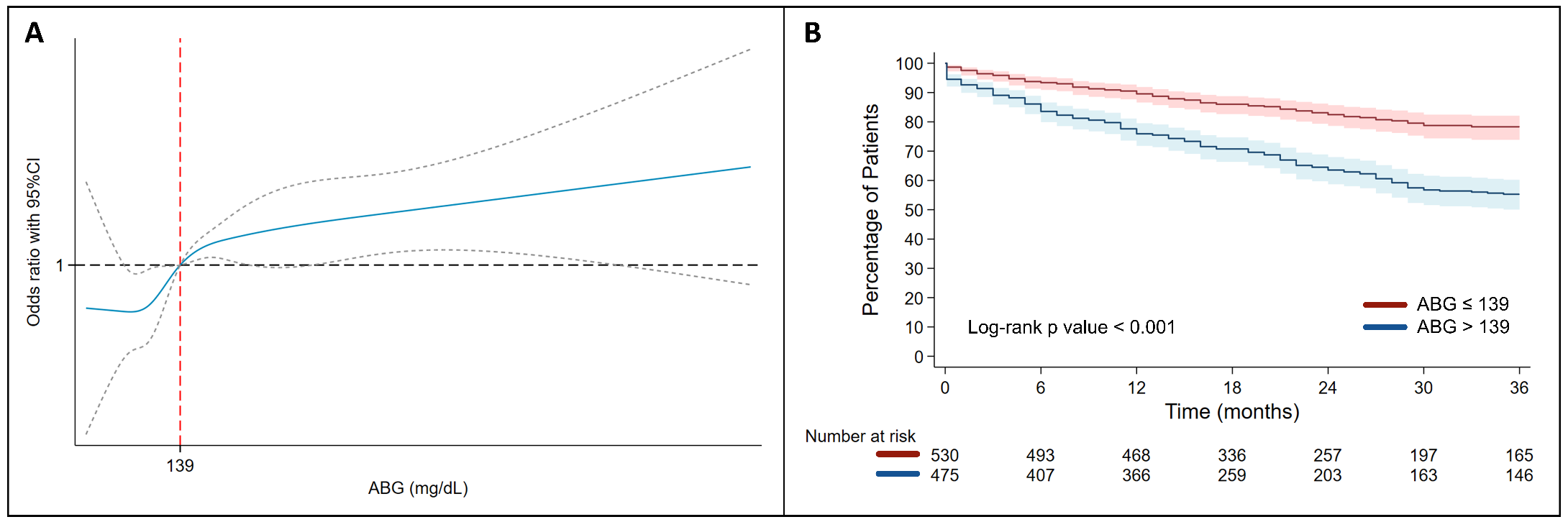
**

Abbreviations: MACEs = major adverse cardiovascular events; ABG = admission blood glucose; ROC = receiver operating characteristic; CI = confidence interval.
